# Evaluating the effects of large marine predators on mobile prey behavior across subtropical reef ecosystems

**DOI:** 10.1002/ece3.5784

**Published:** 2019-11-28

**Authors:** Lindsay M. Phenix, Dana Tricarico, Enrique Quintero, Mark E. Bond, Simon J. Brandl, Austin J. Gallagher

**Affiliations:** ^1^ Beneath the Waves Herndon VA USA; ^2^ Three Seas Program Northeastern University Nahant MA USA; ^3^ Florida International University North Miami FL USA; ^4^ Department of Biological Sciences Simon Fraser University Burnaby BC Canada

**Keywords:** baited remote underwater video stations, predation risk, predator, risk effects, sharks

## Abstract

The indirect effect of predators on prey behavior, recruitment, and spatial relationships continues to attract considerable attention. However, top predators like sharks or large, mobile teleosts, which can have substantial top–down effects in ecosystems, are often difficult to study due to their large size and mobility. This has created a knowledge gap in understanding how they affect their prey through nonconsumptive effects. Here, we investigated how different functional groups of predators affected potential prey fish populations across various habitats within Biscayne Bay, FL. Using baited remote underwater videos (BRUVs), we quantified predator abundance and activity as a rough proxy for predation risk and analyzed key prey behaviors across coral reef, sea fan, seagrass, and sandy habitats. Both predator abundance and prey arrival times to the bait were strongly influenced by habitat type, with open homogenous habitats receiving faster arrival times by prey. Other prey behaviors, such as residency and risk‐associated behaviors, were potentially driven by predator interaction. Our data suggest that small predators across functional groups do not have large controlling effects on prey behavior or stress responses over short temporal scales; however, habitats where predators are more unpredictable in their occurrence (i.e., open areas) may trigger risk‐associated behaviors such as avoidance and vigilance. Our data shed new light on the importance of habitat and context for understanding how marine predators may influence prey behaviors in marine ecosystems.

## Graphical abstract

Assessing behavioral risk effects of marine predators on mobile prey species across varying habitats in Biscayne Bay, FL. Providing new insights into the role marine predators play in influencing prey behavior in a variety of contexts.

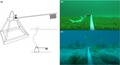

## 1 INTRODUCTION

Top predators are characterized by some of the largest, most enigmatic, and threatened species today on Earth (Hammerschlag & Gallagher, 2017). Often occupying upper trophic tiers, predators can influence prey directly through consumption and also indirectly via the perceived risk of predation. These nonconsumptive effects can drive food‐risk trade‐offs that alter behavior, physiology, and foraging strategies in potential prey (Beauchamp, Wahl, & Johnson, 2007; Heithaus, Frid, Wirsing, & Worm, 2008; Rasher, Hoey, & Hay, 2017). In doing so, predators drive important ecosystem processes that may induce cascading effects throughout entire ecosystems (Estes et al., 2011). Despite the important roles they play in ecosystem dynamics, many populations of large predators are declining rapidly as a result of overexploitation, and habitat loss, among a myriad of other threats (Lennox, Gallagher, Ritchie, & Cooke, 2018).

While effects of apex predators are relatively well studied in terrestrial ecosystems (e.g., Suraci, Clinchy, Dill, Roberts, & Zanette, 2016), their roles in marine systems are generally less understood (e.g., Casey et al., 2017; Sandin et al., 2008). Sharks, for instance, are traditionally considered the de facto top predator in marine ecosystems, and their vulnerabilities to fishing (Gallagher, Kyne, & Hammerschlag, 2012) and general patterns of population decline (e.g., Ferretti, Worm, Britten, Heithaus, & Lotze, 2010) have reinforced the importance of understanding the implications of their removals on marine ecosystems. Often uniformly characterized as apex predators due to their size and trophic position in marine food webs (Heupel, Knip, Simpfendorfer, & Dulvy, 2014; Hussey et al., 2014), sharks may exert strong controlling influences on prey through behaviorally‐mediated, nonconsumptive processes (i.e., predation risk) (Heithaus et al., 2008; Heithaus, Wirsing, Burkholder, Thomson, & Dill, 2009). However, the degree to which sharks actually influence the behavior and physiology of prey species remains understudied and controversial (Casey et al., 2017; Roff et al., 2016; Ruppert, Travers, Smith, Fortin, & Meekan, 2013). Studies have suggested that on coral reefs, herbivorous fish reduce their feeding rates when exposed to a larger, stationary shark decoy (Catano, Barton, Boswell, & Burkepile, 2017; Madin, Gaines, & Warner, 2010; Rizzari, Frisch, Hoey, & McCormick, 2014), but it is unknown whether this acute suppression actually triggers a long‐term reduction in feeding or if it simply redistributes the prey fish to a different area. Similarly, it remains unknown how other sympatric marine teleost predators, such as barracudas (family Sphyraenidae) or morays (family Muraenidae), compare to sharks with regard to their nonconsumptive effects on prey. Nonconsumptive effects would be expected to be particularly prevalent in shallow, open ecosystems where a larger prey item's opportunity for escape from roving, apex predators are limited (Heithaus et al., 2009), thus suggesting a potential effect of habitat complexity.

The lack of a generalizable predator effect (consistency in direction and strength) may be expected in diverse, three‐dimensional ecosystems such as coral reefs where water is clear and opportunities to shelter temporarily are extensive. These habitats provide increased visibility for and detectability of mobile, roving predators. Studies have suggested that in coral reef food webs, reef‐associated sharks and large teleosts occupy similar trophic niches (Bond et al., 2018; Frisch et al., 2016; Roff et al., 2016), which may allow for the detection of generalizable effects of predators on prey or may divert or dilute the nonconsumptive effects of species traditionally considered apex predators on larger prey species. Our knowledge of nonconsumptive effects of marine predators on prey may benefit from examining predator–prey interactions under varying environmental conditions.

An increasingly popular technique for noninvasively assessing the relative abundance and behavior of mobile fish populations, while removing diver bias, is baited remote underwater video (BRUV) surveys (Whitmarsh, Fairweather, & Huveneers, 2017). BRUVs consist of an underwater camera focused on a standardized bait source positioned in the field of view (FOV), with the unit orientated down current from the camera. Individuals attracted to the bait that swim into the FOV are “captured” on camera (Armstrong, Bagley, & Priede, 1992), providing a permanent record of observations that can be reviewed multiple times. This record improves the accuracy of the data and allows for detailed analyses such as those required for examining animal behavior. They have also been used in studies assessing predator–prey relationships (e.g., Klages, Broad, Kelaher, & Davis, 2014) and could be readily used to investigate the potential effects of marine predators on a suite of prey species, across a variety of habitats and conditions.

Here, we used BRUVs to examine the nonconsumptive effects of multiple marine predators on various mobile prey species, across the varying habitats of Biscayne Bay, Florida. We evaluated these predator–prey interactions in three ways: (a) inferring ambient risk in each habitat by quantifying relative predator abundance and foraging activity; (b) assessing habitat‐specific responses of potential prey species by measuring prey arrival (as a proxy for apprehensiveness); and (c) gauging risk‐associated behaviors of prey as well as prey residency at the bait stations (Bond et al., 2019). We hypothesized that (a) predator activity would be greater in complex habitats (Bruno, Stachowicz, & Bertness, 2003; Hutchinson, 1957); (b) prey would take longer to arrive in less complex, more open habitats due to limited shelter opportunities; (c) prey residencies would increase and the number of risk‐associated behaviors would decrease in more complex habitats (Bruno et al., 2003).

## 2 METHODS

### 2.1 Study site

**Figure 1:**
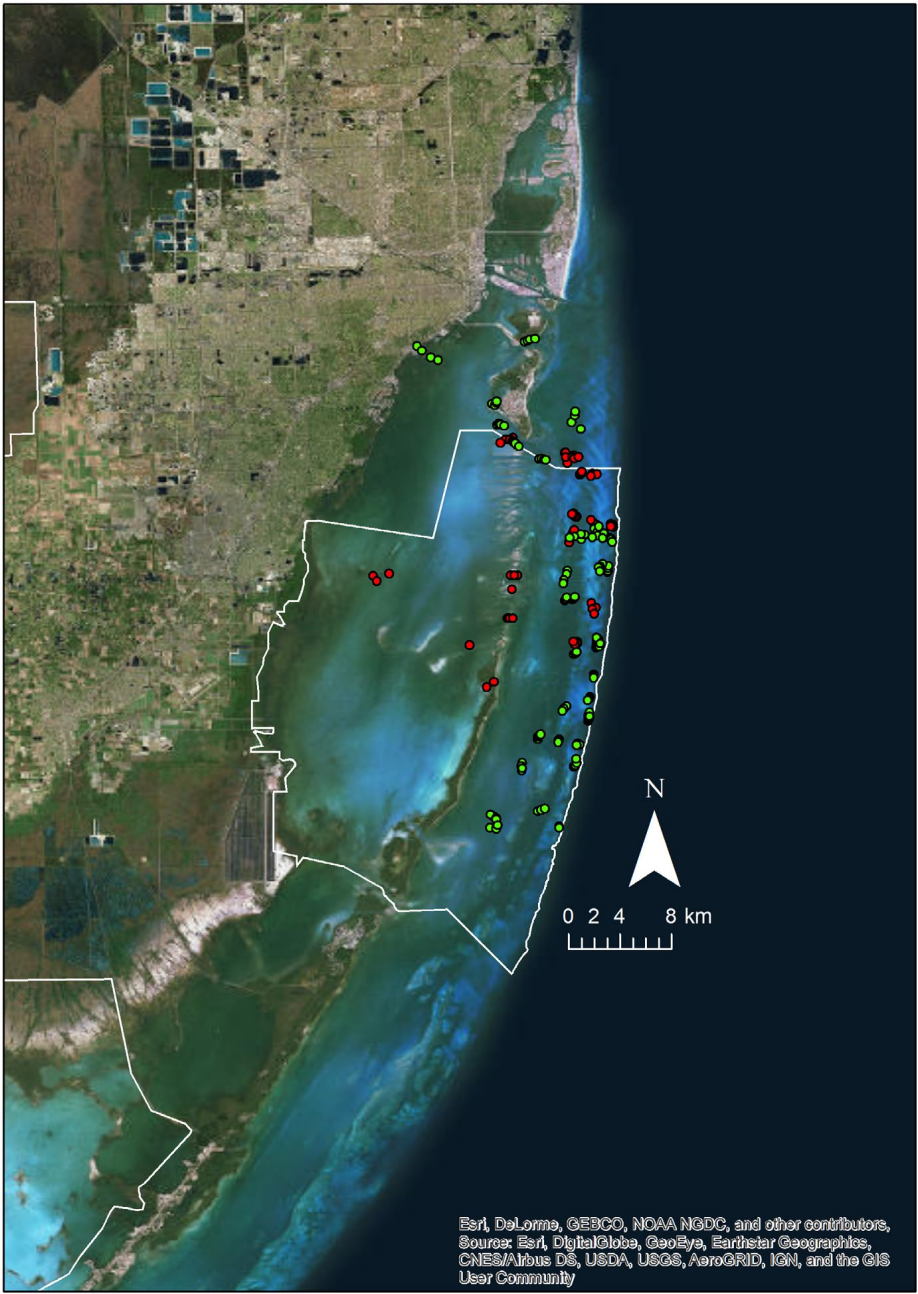
Map of BRUV survey deployments in Biscayne Bay, FL, USA. Red dots = dry season, green dots = wet season. White line represents boundary of Biscayne National Park

This study was conducted from January 21 to August 31, 2017 in the waters of Biscayne Bay, Florida, USA, including within the boundaries of Biscayne National Park (BNP; 25°45′42.05″N, 80°11′30.44″W; Figure 1). This area extends from Key Biscayne to Key Largo and connects to the Florida Reef Tract, the third largest coral reef system worldwide. The area is defined by a mixture of coral reefs, seagrass beds, soft corals, and sand flats. Biscayne Bay is a shallow water lagoon in which a variety of habitats provide important functional, ontogenetic, and trophic value for mangrove and reef‐associated fish, including sharks and rays, as well as sea turtles and marine mammals (Serafy, Valle, Faunce, & Luo, 2007).

### 2.2 Baited Remove Underwater Video (BRUV) surveys

**Figure 2:**
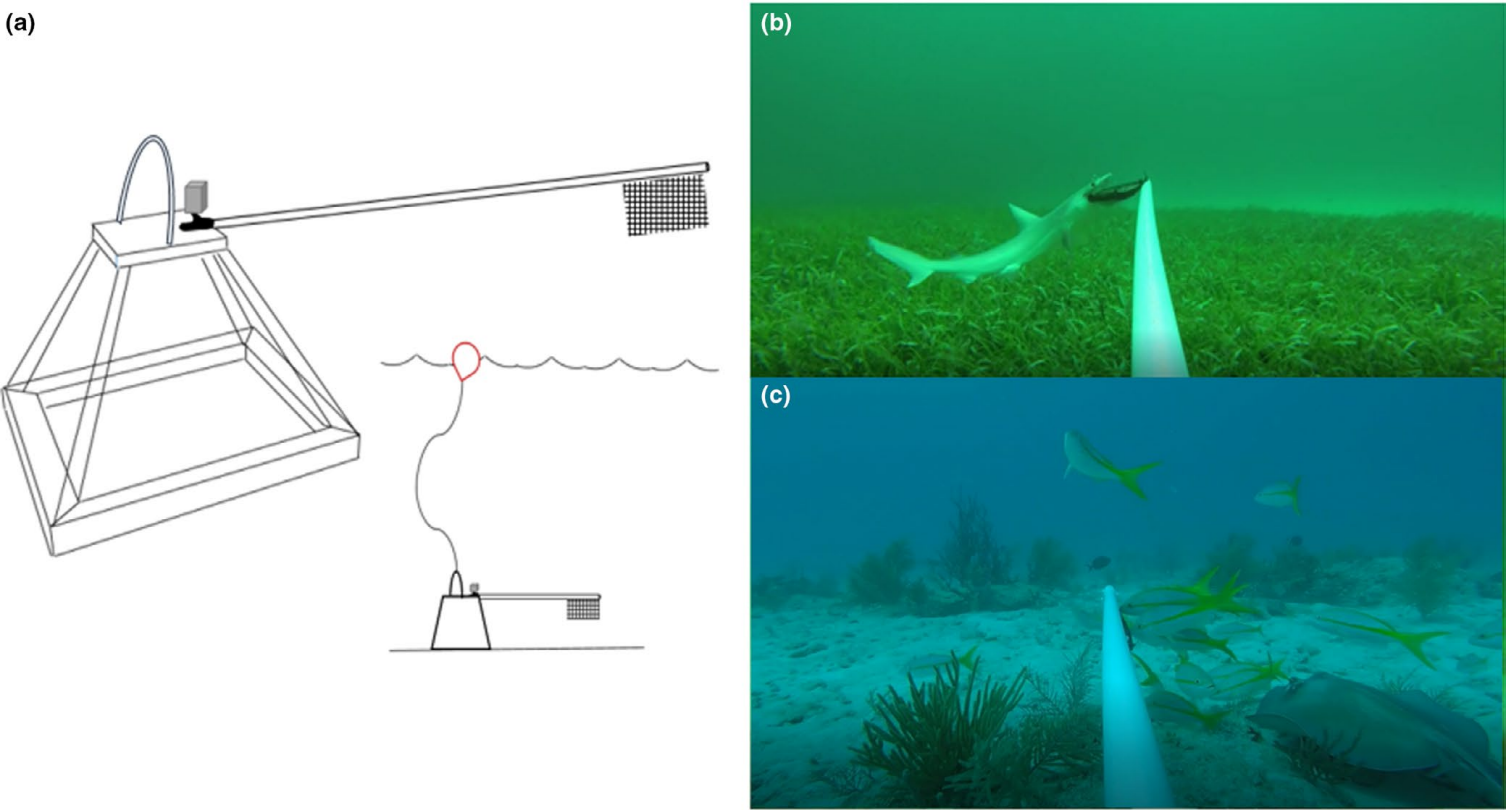
(a) The BRUV assembly; base 74 cm × 74 cm, slant height 72 cm, total height 48cm. (b) Still image captured from BRUV deployment with a bonnethead shark (*Sphyrna tiburo*) in frame. (c) Still image captured from BRUV deployment with schooling yellow snappers (*Ocyurus chrysurus*) and a southern stingray (*Hypanus americanus*) in frame

Predator–prey interactions among and between mobile elasmobranch and teleost communities were assessed throughout Biscayne Bay and Biscayne National Park using baited remote underwater video (BRUV) surveys. Each BRUV consisted of a 48‐cm tall metal pyramid frame with the sides converging at a flat, square platform (Figure 2). Additional weights (two, 0.5 kg dive weights) were added to each BRUV frame to increase stability. Each BRUV was equipped with a 100‐cm PVC bait pole, with a mesh bait bag (150 mm × 200 mm) attached at the end (via zip ties) containing ~450 g of freshly minced Spanish sardines (*Sardinella* spp.). High‐definition action cameras (GoPro Hero and Hero+) were secured to the square platform and positioned to face outward, with the bait bag within the estimated 160° FOV, all lights and flashing sensors on the cameras were deactivated. All footage was shot at 1,080p high‐definition at 30 frames per second.

All BRUVs were deployed from a boat and lowered to the sea floor via 30‐m ropes attached to a visible surface buoy. Deployment depths ranged from 1.3 m to 12.8 m with an average depth of 6.7 m. In‐water free‐divers were occasionally used to navigate the BRUVs away from living corals and to ensure proper orientation on the benthic substrate. BRUVs were deployed in contiguous areas in groups of three to five, spaced ~300–500 m apart, and were allowed to soak for 60 min. Deployments were focused in the following habitat types: coral reef (defined by the presence of coral colonies and structures), sea fan (defined by the presence of patchy sea fans), seagrass (defined by contiguous areas dominated by seagrass), and sand (defined by low‐rugose habitats with open sandy areas). Deployments occurred during daylight hours, between 0800 and 1330 hr. During each round of BRUV deployments, we measured depth and water temperature (°C) using a HANNA handheld probe (Hanna Instruments, HI 98193). Temperature was recorded as a control to account for any possible anomalies; average water temperature was 24.4°C across seasons. We characterized the habitat type as coral reef, sand, sea fan, or seagrass (based on 50% coverage or higher) and whether the site was inside or outside the boundaries of Biscayne National Park (a national park with varying fishing regulations, though it is not a no‐take zone nor a marine reserve).

### 2.3 Video analysis and variables considered

Each 60‐min video was reviewed and analyzed in real time. Analysis began once the BRUV was firmly planted in the benthos (~15–30 s) after deployment. Predators were categorized into three trophic tiers. Upper trophic predators included barracudas (*Sphyraena barracuda*), as well as large bodied (>2 m) mid‐water feeding sharks. Large bodied mid‐trophic predators included large benthic feeding sharks and green moray eels (*Gymnothorax funebris*), while small bodied mid‐trophic predators encompassed small bodied sharks (<2 m) and spotted moray eels (*Gymnothorax moringa*). Groupings were determined based on relative size and the presumed correlating trophic pressures they placed on the ecosystem (Bond et al., 2018; LaymanWinemiller, Arrington, & Jepsen, 2005). Seven common prey families were identified and used to measure habitat risk and risk effects: filefish (family Monacanthidae), grunts (family Haemulidae), jacks (family Carangidae), porgies (family Sparidae), rays (family Dasyatidae & Urotrygonidae), snappers (family Lutjanidae), and triggerfish (family Balistidae). These prey families were chosen due to their observed abundance in the surveyed habitats, and since they reflect a range of consumed prey items for members of the trophic levels listed above. For example, barracuda are known to be important predators of the selected families in our study region (Hansen, 2015). Large shark species found in Biscayne Bay and Florida Bay, such as blacktip (*Carcharhinus limbatus*), bull (*Carcharhinus leucas*), great hammerhead (*Sphyrna mokarran*), and lemon sharks (*Negaprion brevirostris*), retain higher trophic positions than many of the prey families and are known fish predators (Gallagher, Shiffman, Byrnes, Hammerschlag‐Peyer, & Hammerschlag, 2017; Hammerschlag, Luo, Irschick, & Ault, 2012; Matich, Heithaus, & Layman, 2011; Roemer, Gallagher, & Hammerschlag, 2016). Bonnetheads (*Sphyrna turbo*) and Atlantic sharpnose (*Rhizoprionodon terraenovae*) sharks may have varying feeding patterns, but are primarily inshore feeders with diets consisting of teleosts, crustaceans, and cephalopods (Plumlee & Wells, 2016). Similarly, grunts, jacks, and snapper have been found inside the stomachs of nurse sharks in Florida (Castro, 2000). While there is limited data on moray eel diet in our study area, work from other Caribbean areas suggests that they are piscivorous and readily consume snappers or grunts (Randall, 1967; Young & Winn, 2003).

The relative risk of each habitat where a BRUV was deployed was estimated using two predator‐focused variables: (a) predator abundance (maxNb and maxN) and (b) predator foraging activity. Predator abundance was quantified for each trophic grouping (maxNb) by tallying the number of distinctly different individuals, determined by family, sex, size, and markings, observed throughout the entire video duration (Bond et al., 2012). Additionally, a combined predator abundance was taken from each BRUV in the form of maxN, which represents the maximum number of predators, regardless of grouping, present together at one time (Bond et al., 2012). We quantified predator foraging activity rates on the bait bags by recording the number of bites from predators and whether severe damage occurred to the bag (0 = no damage, 1 = severe damage). Bait bags were categorized as “severe damage” if the bag had major lacerations or rips, or if the bag was totally removed from the pole. Nonpredatory fish also have the potential to inflict damage to the bags (i.e., triggerfish), so any instances of damage to the bags from nonpredatory fishes (ascertained via video validation) that could have confounded the detectability of our bait were not included in these analyses.

Potential responses of prey species to ambient predation risk were estimated using arrival times for each prey family (as a proxy for apprehensiveness), as well as evaluating three prey‐focused behaviors (burst swimming, schooling, and bait residency). Arrival time (s) was measured by recording the total elapsed time until the first individual from each prey family arrived on camera. Burst swimming events (defined as a short, rapid swimming behavior away from the frame; Gallagher, Brandl, & Stier, 2016; Gallagher, Lawrence, Jain‐Schlaepfer, Wilson, & Cooke, 2016) and schooling events (defined as instances where groups of five or more conspecific individuals were present; Viscido, Parrish, & Grünbaum, 2005) were recorded for the previously defined prey groups. Bait residency (sec) was evaluated for each replicate as follows: the first fish, regardless of species, to make contact with the bait was monitored until it had moved an estimated three or more body lengths distance from the bait bag (Bond et al., 2019).

### 2.4 Statistical analyses

Because data violated assumptions of normality and homogeneity of variance (confirmed using Shapiro–Wilk's and Levene's tests), we performed a zero‐inflated generalized linear model (GLM) with a negative binomial error distribution and a log‐link function to assess the ambient risk of each habitat, with habitat type and its interaction with predator functional groups specified as the independent variables and predator maxNb as the response variable. Similarly, we performed a GLM with a negative binomial error distribution on prey arrival times, with the response variable being the arrival time of prey species and the independent variables being habitat type, predator maxN, and their interaction. Instances where an individual from a prey family did not appear on the BRUV footage (i.e., not arriving) were excluded from the model. Because this resulted in low replicates for some prey fish species (e.g., rays), we did not specify prey species as an independent variable and assumed that effects of predators are generalized across all prey species. For both GLMs, we used the obtained parameters for predictions and then plotted the predicted values against the raw data to visualize both the obtained patterns and the model fit.

Predator foraging activity and prey behaviors were then visualized using a nonmetric multidimensional scaling ordination (nMDS) based on a Manhattan distance. Furthermore, a PERMANOVA was run on the same distance matrix in order to determine if habitat type, predator maximum abundance, or their interaction affected prey behavior. Finally, we analyzed correlations between predator foraging and prey risk‐associated behaviors for each habitat using a set of Spearman rank correlation analyses. All statistical analyses were performed using R Studio (R Core Team).

## 3 RESULTS

A total of 194 deployments were made, within a total survey area of ~15 km^2^. Of these, 37 deployments were discarded due to the BRUV tipping over in heavy current or poor visibility, leaving a total of 157 videos (*n* = 157) that were used in analyses (Table 1). A total of 184 predators were recorded by the BRUVs throughout the sampling period (Table 2). Of those predators, 80 individual elasmobranchs from eight species (7 shark species, 1 ray species) were recorded, in addition to 88 barracuda and 16 moray eels. There were limited seasonal differences in maximum predator abundances (maxN) and prey arrival times across habitats, except for seagrass beds, where maximum predator abundance was substantially higher in the wet season (0.690 ± 0.0.123 individuals, mean ± *SE*) than in the dry season (0.091 ± 0.063). In fact, no barracudas or large bodied mid‐trophic predators were observed in seagrass habitats during the dry seasons. However, prey arrival times in seagrass beds did not differ between the two seasons.

**Table 1:** BRUV deployments by season and habitat type

Habitat	Season	
	Dry (January–April)	Wet (May–December)
Coral reef	4	15
Sea fan	9	34
Seagrass	22	30
Sand	16	27

**Table 2:** Summary of predatory species observed on BRUVs in the present study

Upper trophic		Large mid‐trophic		Small mid‐trophic	
Barracuda (*Sphyraena* sp.)	88	Green Moray (*Gymnothorax funebris*)	4	Atlantic Sharpnose (*Rhizoprionodon terraenovae*)	14
Blacktip (*Carcharhinus limbatus*)	3	Nurse (*Ginglymostoma cirratum*)	22	Blacknose (*Carcharhinus acronotus*)	3
Bull (*Carcharhinus leucas*)	2	Sawfish (*Pristis pectinata*)	1	Bonnethead (*Sphyrna turbo*)	34
Great Hammerhead (*Sphyrna mokarran*)	1			Spotted Moray (*Gymnothorax moringa*)	12
Total	94		27		63

**Figure 3:**
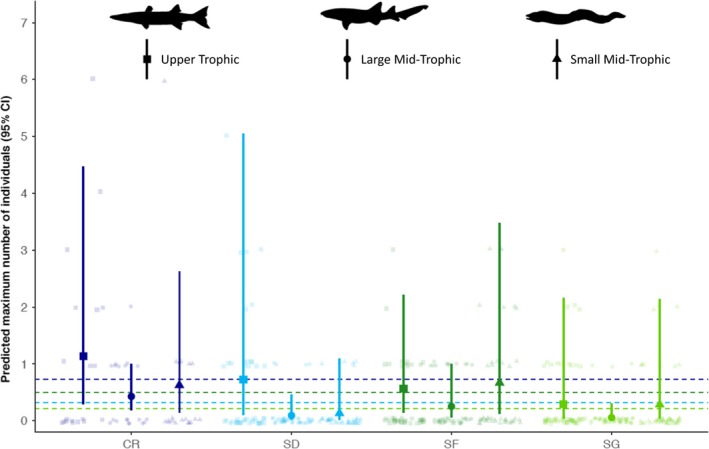
Mean predicted predator abundance (±95% confidence intervals) from a zero‐inflated negative binomial GLM across four habitat types: coral reef (CR), sand (SD), sea fan (SF), and seagrass (SG). Predicted predator abundance values, as well as mean predicted abundance by habitat (dashed lines) are overlaid on top of raw observational data

**Figure 4:**
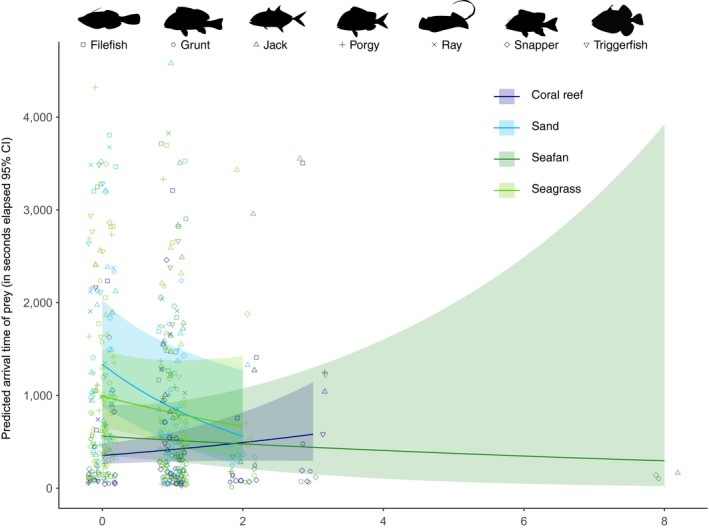
Predicted mean prey arrival time (*y*‐axis) as a function of maximum combined predator abundance (*x*‐axis) across four habitat types based on a negative binomial GLM. Predicted fits (±95% confidence intervals) are overlaid on top of raw observational data of seven prey families across four habitat types. CR, coral reef; SD, sand; SF, sea fan; SG, seagrass

Predator abundances (maxNb) were significantly different among habitat types, with coral reefs having the highest average maximum number of predators per deployment (2.21 ± 2.04), followed by sea fan habitats, sand, and seagrass habitats (Table 3, Table 4). Predictions from the GLM further suggest an interaction effect between trophic level grouping and habitat. Coral reefs had the greatest mean abundance of upper trophic and large bodied mid‐trophic predators, whereas sea fan habitats had the greatest mean abundances of small bodied mid‐trophic predators (Figure 3). Prey arrival times were significantly influenced by the interactive effects of habitat and the cumulative maximum number of predators (maxN) (Table 5). Grunts, porgies, and snappers arrived comparatively early at the BRUV deployments, while stingrays arrived substantially later. The GLM revealed that the effect of maximum predator numbers in sand, sea fan, and seagrass habitats are negative and significantly different from effects of predators on coral reefs, where cumulative predator maximum number and prey arrival time were positively correlated. This is further supported by the predictions from the model, which show a steep negative relationship in sand and seagrass habitats, a nearly flat but slightly negative relationship in sea fan habitats and a positive relationship for coral reefs (Figure 4).

**Table 3:** Mean predator abundance per BRUV deployment across the four habitat types (coral reef, sand, sea fan, and seagrass), decomposed into the different trophic levels and their combined abundance (MaxNb)

	Upper trophic	Large mid‐trophic	Small mid‐trophic	Max Nb
Coral reef	1.16 (±0.384)	0.474 (±0.140)	0.579 (±0.318)	2.21 (±2.04)
Sand	0.674 (±0.169)	0.093 (±0.045)	0.140 (±0.053)	0.907 (±1.231)
Sea fan	0.581 (±0.245)	0.256 (±0.067)	0.721 (±0.206)	1.56 (±1.94)
Seagrass	0.346 (±0.095)	0.058 (±0.033)	0.288 (±0.092)	0.692 (±1.15)

**Table 4:** Summary results from a zero‐inflated negative binomial generalized linear model used to test the effects of habitat type on predator abundance (maxNb) by trophic level

	Coefficients	Estimate	*SE*	*Z* value	Pr(>|*z*|)
	Intercept (coral reef:large mid‐trophic)	−9.041	0.43	−21.03	***
	Sand	−1.598	0.685	−2.33	*
	Sea fan	−0.659	0.555	−1.19	ns
	Seagrass	−2.137	0.739	−2.89	**
CR	Upper trophic	0.886	0.551	1.61	ns
	Small mid‐trophic	0.252	0.591	0.43	ns
SD	Upper trophic	1.993	0.594	3.35	***
	Small mid‐trophic	0.435	0.697	0.62	ns
SF	Upper trophic	0.82	0.443	1.85	.
	Small mid‐trophic	1.016	0.434	2.34	*
SG	Upper trophic	1.766	0.667	2.65	**
	Small mid‐trophic	1.594	0.675	2.36	*

Note

Significant codes: ‘***’ 0.001 ‘**’ 0.01 ‘*’ 0.05 ‘.’ 0.1.

**Table 5:** Summary results of a negative binomial generalized linear model of the effects of habitat type on maximum combined predator abundance (maxN)

Coefficients	Estimate	*SE*	*Z* value	Pr (>|*z*|)
Intercept (coral reef)	5.865	0.156	37.6	***
Sand	1.331	0.172	7.73	***
Sea fan	0.463	0.174	2.66	**
Seagrass	1.035	0.168	6.18	***
maxN	0.167	0.100	1.67	ns
Sand: maxN	−0.601	0.141	−4.26	***
Sea fan: maxN	−0.247	0.114	−2.17	*
Seagrass: maxN	−0.366	0.130	−2.82	**

**Figure 5:**
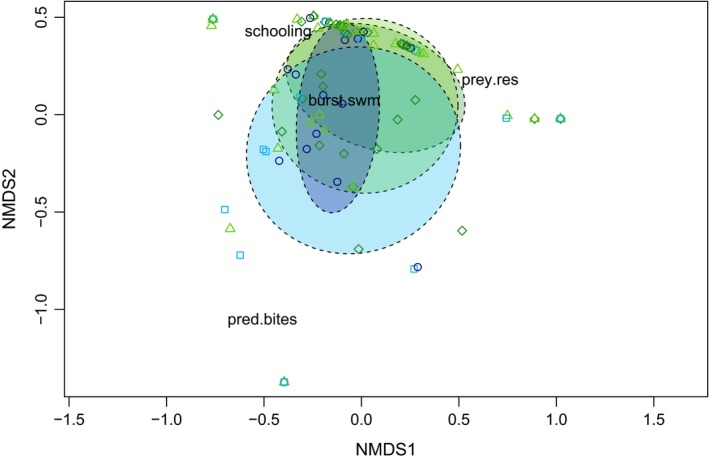
Multidimensional ordination of predator foraging activity (predator bites and bait damage) and prey risk‐associated behaviors (burst swimming, schooling, and prey residency) across four habitat types. Colors match the previously used habitat‐specific colors

**Figure 6:**
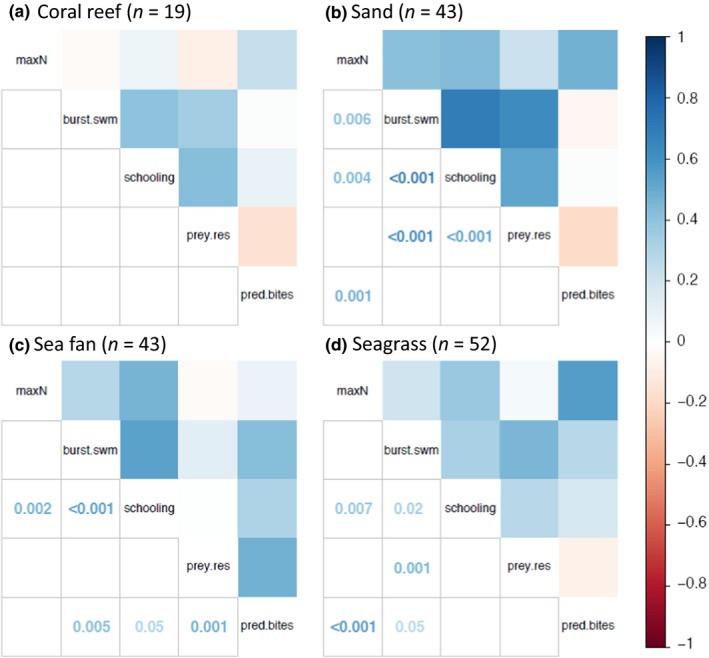
Correlation plot of prey risk behaviors (burst swimming, schooling, and prey residency) compared to predator foraging activity (bites and damage) across four habitat types

The nMDS ordination of both predator foraging activity (i.e., number of bites) and prey behavior in response to habitat type showed little variation among habitats (Figure 5). Generalized predator foraging activity was not significantly influenced by any habitat type, although BRUVs deployed on coral reefs experienced the highest average number of predatory bites (2.211 ± 3.441 bites, mean ± SE) and instances of severe damage to the bait bag (0.263 ± 0.452 instances, mean ± SE). Prey burst swimming (4.579 ± 7.932 events) and schooling events (6.053 ± 4.801 events) also had the highest average occurrences on coral reefs when compared to sand, sea fans, and seagrass habitats (Table 6). Average prey residency at the bait was the greatest in sea fan habitats (32.211 ± 32.527 s). The PERMANOVA to test the explanatory power of habitat, predator maximum number, and their interaction on different behaviors, albeit revealing a significant habitat effect (*p* = .001), only explained ~10% of the variation in the data and no effect of predator maximum number or its interaction with habitat was observed. The Spearman rank correlation test showed significant correlations between predator and prey behaviors in sand, seagrass, and sea fan habitats, but not on coral reefs (Figure 6). Schooling behavior was the only one to show a positive relationship with predator maximum numbers across sand, seagrass, and sea fan habitats.

**Table 6:** Mean predator foraging activity (bites and severe damage) and prey response behavior (burst swimming, schooling, and residency) across four habitat types

	Predator bites	Severe damage	Burst swimming	Schooling	Prey residency
Coral reef	2.211 (±3.441)	0.263 (±0.452)	4.579 (±7.324)	6.052 (±4.801)	24.316 (±18.973)
Sand	0.791 (±1.684)	0.070 (±0.259)	0.698 (±3.377)	1.395 (±2.555)	8.814 (±17.14)
Sea fan	1.558 (±4.078)	0.136 (±0.351)	1.605 (±3.13)	4.628 (±4.232)	32.211 (±32.527)
Seagrass	0.865 (±2.360)	0.096 (±0.298)	0.745 (±1.741)	2.980 (±3.906)	20.192 (±27.652)

## 4 DISCUSSION

Predator–prey interactions can structure marine habitats by actively changing habitat use, foraging behaviors, and food‐web dynamics (Morosinotto, Thomson, & Korpimäki, 2010). We predicted that prey fishes would be more apprehensive and thus arrive later in the field of view of the BRUV in habitats with increased predator abundance and vice versa in those with fewer predators. Our results suggest that this pattern held true only for coral reefs, where predator numbers appeared to have a negative effect on prey arrival, while in all other habitats, the two variables were positively correlated. While coral reefs offer increased structural complexity and refuge for prey, they also increase potential predation risk by obscuring prey fish's field of view (Bond et al., 2019). These components of the habitat may provide predators with a functional advantage when hunting, thereby creating a more dangerous environment and increasing prey vigilance in these areas. Thus, the interaction between habitat features and the probability of predator detection and successful escape can result in altered prey risk‐associated behaviors and vigilance (Heithaus et al., 2009). It has been recently argued that predators may exact greater influences on prey behavior where predation risk is predictable (Creel, 2018). While we did not measure predictability of predation risk in our study, abundance of predators in certain habitats, a potential proxy for exposure, may have resulted in a pro‐active response of apprehensiveness toward the bait, although this remains speculative.

Predators are known to match prey distributions on small scales when prey is abundant (Heithaus & Dill, 2006), and, as observed in the present study, coral reefs generally contain high numbers of piscivores (Hixon & Beets, 1993), which can inversely affect prey abundance on reefs (Beukers‐Stewart, Beukers‐Stewart, & Jones, 2011). On average, grunts and snappers arrived on coral reefs and in sea fans long before any predators. Whether predation risk is “predictable” or chronic on coral reefs remains unknown, but our findings offer an interesting potential link to the predicted food‐risk effects as described in the “control of risk” hypothesis (Creel, 2018).

Animals often express their antipredator‐behaviors in high risk situations that are brief and infrequent (Lima & Bednekoff, 1999). These acute “reactive” responses are linked to areas of unpredictable predation risk (Creel, 2018). We hypothesized that potential behavioral risk effects might be highest in open areas. Interestingly, we observed faster prey arrival times in more open, homogenous habitats such as sea fans, sandy areas, and seagrass. The lack of resources in these open, plain habitats may have rendered our BRUVs a more attractive source of food, resulting in both prey and predators arriving sooner; in our study, we found that grunts and snappers were much quicker to arrive to a habitat where predators were more abundant (Nagelkerken & Velde, 2004). It is also possible that these open and homogenous habitats provide increased escape routes to prey if needed, thus making them worth the “risk.” Additionally, since predators are often transient in these habitats (Hammerschlag, Morgan, & Serafy, 2010), attacks may be less predictable. Therefore, our observed patterns for behavioral effects in these habitats may stem from a combination of resource provisioning and unpredictability of predation risk.

In general, juvenile and small bodied sharks (i.e., small mid‐trophic predators) can be found in shallow waters to minimize their own predation risk (Guttridge et al., 2012; Heupel et al., 2014). More than half of the sharks captured on the BRUVs were species that reach maximum sizes of <2 m. While it stands to reason that smaller predators induce a weaker response in prey than larger conspecifics or species (due to gape limitations), smaller mesopredators (hawkfish, *Parrachirrhites arcatus*) have been found to have equal nonconsumptive effects compared to larger conspecifics (Gallagher, Brandl, et al., 2016; Gallagher, Lawrence, et al., 2016). Most predators (regardless of trophic grouping) in our videos did not stay for prolonged periods of time and, as such, they represent an acute, but relatively inconsistent, pulsed source of predation risk. Finally, some small species (e.g., bonnetheads) may also have limited effects on prey because both juveniles and adults primarily feed on crabs, lobsters, and cephalopods (Bethea et al., 2007).

The extrapolation of our results beyond our study design is hindered by several caveats. Firstly, we do not know whether arrival times are truly a consequence of perceived predation risk or if they are a function of varying densities of individuals which could not be controlled. We also did not measure water currents at each of our BRUV stations, which could have affected the bait dispersal at different rates, thus changing detection potential by prey species. Furthermore, our statistical power was weakened by poor visibility (resulting in the exclusion of 37 replicates) and a category 5 hurricane, which ended data collection a bit early and thus prevented extended sampling. In future studies, dusk or night time deployments should be added to observe predator–prey interactions after dark, which may be especially important for sharks on coral reefs (Hammerschlag et al., 2017).

The role of “apex”‐predators on reefs has been brought into question in recent years (see Roff et al., 2016). While we caution overextending the results of this study to other regions, our data suggest that predators regardless of their trophic position do not significantly control mobile prey behavior on short temporal scales, across habitats. Instead, a habitat‐specific response to a consistent signal of mobile predators on reefs may result in proactive prey vigilance and subtle food‐risk trade‐offs. Specifically, less complex habitats where predators are known to patrol yet remain temporally unpredictable in their occurrence due to limited numbers and potentially wider activity areas may induce different reactive behavioral effects such as schooling and burst swimming, which, when extended over larger time scales, could have metabolic and fitness‐level impacts on prey. Taken together, these results suggest that context is important when trying to disentangle the effects of top predators on prey in costal marine habitats, and future studies should examine the interactions between mobile predators and habitat in order to link predation risk theory to observations.

## CONFLICT OF INTEREST

The authors declare no competing interests.

## AUTHOR CONTRIBUTIONS

A. J. G., M. E. B., and S. J. B. conceived and designed the study. L. M. P., D. T., E. Q., and A. J. G. conduced the field work. L. M. P. and S. J. B. performed the analyses. All authors contributed to writing the manuscript and gave approval.

## Data Availability

All data are deposited and available in Dryad at the following address https://doi.org/10.5061/dryad.wm37pvmh5.
